# Elderly Male with a Painful Red Eye

**DOI:** 10.5811/cpcem.6593

**Published:** 2026-07-28

**Authors:** Diala Dahdal, Ahmed Nama, Wenyu Deng, Barry Hahn

**Affiliations:** * Hadassah University Medical Center-Ein Kerem, Department of Emergency Medicine, Jerusalem, Israel; † Hebrew University School of Medicine, Faculty of Medicine, Jerusalem, Israel; ‡ State University of New York, Downstate Medical Center, Department of Ophthalmology, New York, New York; § Staten Island University Hospital, Department of Emergency Medicine, New York, New York

**Keywords:** corneal perforation, emergency department, corneal transplant, case report

## Abstract

**Case Presentation:**

An 83-year-old man with a history of left corneal transplant presented to the emergency department with several days of left eye pain, redness, and tearing. Visual acuity was 20/50 in the right eye and bare light perception in the left eye. Intraocular pressure was 15 millimeters fo mercury in the right eye and unobtainable in the left. The left conjunctiva and sclera were injected, and slit-lamp examination revealed a full-thickness corneal perforation with corneal haze in the transplanted cornea. We placed a rigid eye shield, provided systemic analgesia, kept the patient nil per os, and obtained urgent ophthalmology consultation.

**Discussion:**

Corneal perforation is a full-thickness corneal defect that disrupts globe integrity and allows aqueous leakage. It may result from infection, ocular surface disease, autoimmune disorders, trauma, or prior keratoplasty, with graft–host junction instability representing a key risk factor. Diagnosis is clinical, based on visible perforation, a positive Seidel test, or anterior chamber shallowing.

## CASE PRESENTATION

An 83-year-old male with a history of left corneal transplant presented to the emergency department (ED) with left eye pain, redness, and tearing for several days. Visual acuity was 20/50 in the right eye (OD) and bare light perception in the left eye (OS). Intraocular pressure was 15 mm Hg OD and unobtainable OS. We initially attempted tonometry before the corneal perforation was fully appreciated. Once we identified a visible full-thickness corneal perforation, further measurement was deferred because of concern for worsening ocular injury. The left conjunctiva and sclera were injected. The left cornea had a full-thickness perforation with haziness (Image).

## DISCUSSION

Corneal perforation is a full-thickness corneal defect that disrupts globe integrity and allows aqueous leakage. It results from progressive stromal and collagen breakdown, leading to corneal tissue loss and aqueous leakage. Common causes include infection, ocular surface disease, autoimmune disorders, trauma, or prior corneal transplantation. The incidence of corneal perforation in the ED is 0.67 per 1,000,000 people.^1^ Patients typically present with acute pain, photophobia, and blurry vision. Diagnosis is clinical and can be made by identifying a visible perforation, a positive Seidel test (where fluorescein dye reveals aqueous leakage as a streaming dilution of the dye), or a shallowing of the anterior chamber depth relative to the unaffected eye, suggesting loss of aqueous humor.^2^

In the ED, corneal perforation should be managed as an open-globe injury. Physicians should avoid pressure on the eye, place a rigid eye shield, control pain, and obtain urgent ophthalmology consultation for definitive management. Computed tomography can be considered if an intraocular foreign body is suspected. Definitive management is guided by the size and location of the defect, which ranges from tissue adhesive for small perforations to corneal grafting for larger or more complex cases. Long-term vision may be limited by scarring, graft failure, irregular astigmatism, or glaucoma.^3^

*CPC-EM Capsule*

What do we already know about this clinical entity?

*Corneal perforation is a rare but vision-threatening emergency that may be subtle and requires rapid recognition to prevent permanent vision loss.*

What is the major impact of the image(s)?

*This image highlights a visible corneal perforation, an uncommon but critical finding that emergency physicians must recognize immediately.*

How might this improve emergency medicine practice?

*Early recognition of visible corneal perforation can expedite ophthalmology consultation and help avoid contraindicated tonometry.*

## Figures and Tables

**Image.**
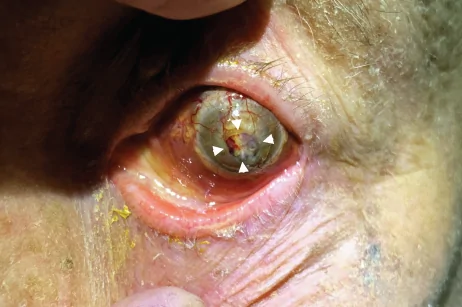
Photograph of the left eye showing opacified cornea with circumferential neovascularization. The white arrowheads highlight a central full-thickness corneal perforation.
